# A Rare Fungal Species, *Quambalaria cyanescens*, Isolated from a Patient after Augmentation Mammoplasty – Environmental Contaminant or Pathogen?

**DOI:** 10.1371/journal.pone.0106949

**Published:** 2014-10-20

**Authors:** Xin Fan, Meng Xiao, Fanrong Kong, Timothy Kudinha, He Wang, Ying-Chun Xu

**Affiliations:** 1 Department of Clinical Laboratory, Peking Union Medical College Hospital, and Graduate School, Peking Union Medical College, Chinese Academy of Medical Sciences, Beijing, China; 2 Centre for Infectious Diseases and Microbiology Laboratory Services, Westmead Hospital, Westmead, New South Wales, Australia; 3 Charles Sturt University, Orange, New South Wales, Australia; Nanjing Agricultural University, China

## Abstract

Some emerging but less common human fungal pathogens are known environmental species and could be of low virulence. Meanwhile, some species have natural antifungal drug resistance, which may pose significant clinical diagnosis and treatment challenges. Implant breast augmentation is one of the most frequently performed surgical procedures in China, and fungal infection of breast implants is considered rare. Here we report the isolation of a rare human fungal species, *Quambalaria cyanescens*, from a female patient in China. The patient had undergone bilateral augmentation mammoplasty 11 years ago and was admitted to Peking Union Medical College Hospital on 15 September 2011 with primary diagnosis of breast infection. She underwent surgery to remove the implant and fully recovered thereafter. During surgery, implants and surrounding tissues were removed and sent for histopathology and microbiology examination. Our careful review showed that there was no solid histopathologic evidence of infection apart from inflammation. However, a fungal strain, which was initially misidentified as “*Candida tropicalis*” because of the similar appearance on CHROMagar *Candida*, was recovered. The organism was later on re-identified as *Q. cyanescens*, based on sequencing of the rDNA internal transcribed spacer region rather than the D1/D2 domain of 26S rDNA. It exhibited high MICs to 5-flucytosine and all echinocandins, but appeared more susceptible to amphotericin B and azoles tested. The possible pathogenic role of *Q. cyanescens* in breast implants is discussed in this case, and the increased potential for misidentification of the isolate is a cause for concern as it may lead to inappropriate antifungal treatment.

## Introduction

Human beings live in a fungal-rich and fungal-diverse environment. Some emerging less common fungal pathogens are known environmental species (from soil, plants, insects, medical facilities, wastes or other outdoor or indoor environments). These fungal organisms are generally of low virulence and some may exhibit natural antifungal drug resistance [1]–[3], which presents clinical diagnosis and treatment challenges [4]–[6]. While some of the fungal infections can be diagnosed easily, in particular if isolated from blood, cerebrospinal fluid, etc., and with clear infection clinical signs, others present challenges in understanding their role in certain infections. Here we report an interesting case to highlight the challenges clinical pathologists and medical doctors faced in the decision making process of a case involving a rare fungus.

The *Quambalariaceae* is a family of fungi in the class *Exobasidiomycetes*. The family contains the single genus *Quambalaria*, which contains five species, including *Quambalaria cyanescens*, *Q. coyrecup*, *Q. eucalypti*, *Q. pitereka* and *Q. simpsonii* [7]–[9]. The first *Q. cyanescens* strain was isolated from human skin (strain no. CBS 357.73, type strain of the species) and reported as *Sporothix cyanescens* by de Hoog *et al.* in 1973 (Table 1) [10]. In 1987, Moore *et al.* erected the genus *Cerinosterus*, reset the previous *S. cyanescens* into this new genus and renamed it as *Cerinosterus cyanescens* [8]. However, a later study by analysis of partial large subunit (LSU)-rDNA sequences and the nutritional profile revealed that *C. cyanescens* was a close relative of *Microstroma juglandis*, but differed from other species within the genus *Cerinosterus* [11]. To resolve this problem, Sigler *et al.* established the new genus *Fugomyces*, and designated *C. cyanescens* as *Fugomyces cyanescens* [11], [12]. Recently, phylogenetic studies conducted by de Beer *et al.* have reassigned this species in the family *Quambalariaceae* as *Q. cyanescens*, based on the analysis of internal transcribed spacer (ITS) region and LSU sequences combined with ultrastructural characteristics [8].

**Table 1:** Summary of *Q. cyanescens* isolates from this study, published literatures or GenBank, and genetic comparison within *Q. cyanescens* species and to selected strains of other *Quambalaria* species.

Strain	Country	Origin	ITS		D1/D2		Reference
			GenBank accession no.	Identity (%*)	GenBank accession no.	Identity (%*)	
***Q. cyanescens*** ** Type strain**							
CBS 357.73	Netherlands	Human skin	DQ119135.1; DQ317622.1	Reference sequence	DQ317615.1; AM261925.1	Reference sequence	[8], [10], [12]
***Q. cyanescens*** ** Human source isolate**							
11PU348	China	Implants	KF953496.1	576/580 (99.3)	KF953497.1	600/600 (100.0)	This study
***Q. cyanescens*** ** Environmental isolates**							
IMI298177	Australia	Plant	AJ535500.1	580/580 (100.0)	NA	NA	Unpublished
IMI178848	Australia	Plant	AJ536610.1	573/575 (99.7)	NA	NA	Unpublished
MK742	Turkey	Beetle	AM261920.1	579/580 (99.8)	AM261920.1	576/576 (100.0)	[12]
MK808	Syria	Beetle	AM261921.2	580/580 (100.0)	NA	NA	[12]
MK1710	Bulgaria	Beetle	AM261922.2	580/580 (100.0)	NA	NA	[12]
CCF3527 = MK617	Hungary	Beetle	AM261923.2	557/559 (99.6)	AM261923.2	576/576 (100.0)	[12]
MK1617	Spain	Beetle	AM261924.2	555/556 (99.8)	NA	NA	[12]
SW326	Unknown	Unknown	NA	NA	AY234900.1	313/313 (100.0)	[38]
CF3526	Czech	Beetle	DQ119134.1	580/580 (100.0)	DQ119136.1	550/552 (99.6)	[12], [39]
CBS 876.73	Australia	Plant	DQ317623.1	578/579 (99.8)	DQ317616.1	601/601 (100.0)	[8]
WAC12952	Australia	Beetle	DQ823419.1	579/579 (100.0)	DQ823440.1	561/561 (100.0)	[9]
WAC12954	Australia	Beetle	DQ823420.1	579/579 (100.0)	DQ823442.1	561/561 (100.0)	[9]
WAC129555	Australia	Beetle	DQ823421.1	573/579 (99.0)	DQ823441.1	561/561 (100.0)	[9]
WAC12953	Australia	Beetle	DQ823422.1	574/580 (99.0)	DQ823443.1	560/561 (99.8)	[9]
BRIP48396	Australia	Beetle	EF444874.1	579/580 (99.8)	NA	NA	[39]
BRIP48398	Australia	Beetle	EF444875.1	579/581 (99.7)	NA	NA	[39]
BRIP48403	Australia	Beetle	EF444876.1	579/579 (100.0)	NA	NA	[39]
U16	USA	Beetle	HF569147.1	559/559 (100.0)	NA	NA	Unpublished
U105	USA	Beetle	HF569150.1	556/556 (100.0)	HF569150.1	277/277 (100.0)	Unpublished
U110	USA	Beetle	HF569153.1	559/559 (100.0)	HF569153.1	277/277 (100.0)	Unpublished
U121	USA	Beetle	HF569155.1	577/577 (100.0)	NA	NA	Unpublished
U161	USA	Beetle	HG421947.1	553/556 (99.5)	HG421947.1	277/277 (100.0)	Unpublished
U163	USA	Beetle	HG421948.1	553/556 (99.5)	HG421948.1	277/277 (100.0)	Unpublished
U182	USA	Beetle	HG421949.1	556/559 (99.5)	HG421949.1	277/277 (100.0)	Unpublished
CCF4578	USA	Beetle	HG421950.1	556/556 (100.0)	HG421950.1	277/277 (100.0)	Unpublished
U144a	USA	Beetle	HG421951.1	556/556 (100.0)	HG421951.1	277/277 (100.0)	Unpublished
U100	USA	Beetle	HG421952.1	559/559 (100.0)	HG421952.1	277/277 (100.0)	Unpublished
CCF4580	USA	Beetle	HG421953.1	559/559 (100.0)	HG421953.1	277/277 (100.0)	Unpublished
CCF4582	USA	Beetle	HG421954.1	577/577 (100.0)	NA	NA	Unpublished
CCF4583	USA	Beetle	HG421955.1	559/559 (100.0)	NA	NA	Unpublished
QY229	China	Rice	HM013823.1	570/574 (99.3)	NA	NA	[40]
AUMC6293	Egypt	Air	JQ425376.1	576/580 (99.3)	NA	NA	Unpublished
AUMC6294	Egypt	Citrus juice	JQ425382.1	576/580 (99.3)	NA	NA	Unpublished
**Other ** ***Quambalaria*** ** species**							
CBS124772 (*Q. simpsonii*)	Australia	Plant	GQ303290.1	575/601 (96.3)	GQ303321.1	601/601 (100.0)	[7]
CMW1101 (*Q. eucalypti*)	South Africa	Plant	DQ317625.1	568/601 (94.5)	DQ317618.1	600/601 (99.8)	[8]
CMW6707 (*Q. pitereka*)	Australia	Plant	DQ317627.1	569/598 (95.2)	DQ317620.1	598/601 (99.5)	[8]
WAC12947 (*Q. coyrecup*)	Australia	Plant	DQ823444.1	560/603 (92.9)	DQ823444.1	556/561 (99.1)	[9]

Abbreviations: ITS, ribosomal DNA internal transcribed spacer region; D1/D2, D1/D2 domain of the 26S ribosomal DNA; NA, not available.

*Refers to identity of the ITS region or D1/D2 domain sequences between type strain CBS 357.73 and other isolates.

*Q. cyanescens* is one of the rare clinical basidiomycetous pathogens. Most of *Q. cyanescens* isolated from the humans were reported in the 1990s, including pseudoepidemic nosocomial pneumonia cases reported in a US hospital [13], a possible pulmonary case in a heart transplant patient [14] and potential fungemia in lymphoma patients [11]. However, none of these published human-related cases deposited convincing molecular data.

In this case study, we report the mycology and molecular characteristics of a *Q. cyanescens* isolate from a 43 year-old female who previously received injected augmentation mammoplasty, and discuss the possible pathogenic role of the organism.

## Methods

### 1. Ethics statement

The present case was from China Hospital Invasive Fungal Surveillance Net (CHIF-NET) study. Study protocol was approved by the Human Research Ethics Committee of Peking Union Medical College Hospital (No. S-263), and written consent was obtained from the patient.

### 2. Clinical case

A 43-year old woman was admitted to the Plastic Surgical Department of Peking Union Medical College Hospital on 15 September 2011 because of left breast pain, with symptoms of redness and swelling. She had previously undergone bilateral injected augmentation mammoplasty around 11 years ago in Fujian Province, China.

The woman was in good health status except for the inflammation of the breast and did not report any other major disease in her clinical history. The blood test results were all within normal values. Clinical examination showed that she was afebrile and no ulceration was present in her left breast. Primary diagnosis was made as left breast infection. Surgical operation was performed to take out the bilateral implants as per patient's request. However, no microbiological examination was done before surgery.

During surgery to remove the implants, it was noted that the yellow-brown semisolid implant had spilled and was mixed with unknown granule, and also there was damage in the mammary tissues. Partial implants and surrounding tissue were sent for histopathologic and microbiological laboratory examination. After surgery, cefmetazole (IV, 1 g bid) was given, combined with metronidazole (IV, 0.915 g, q12h) for 7 days. The patient fully-recovered and was subsequently discharged on 24 September 2011 before the microbiology laboratory results were finalized. She didn't receive any antimicrobial or antifungal treatment since then, nor were any relapses reported at the 12- and 24-month follow-up visits.

### 3. Initial laboratory examinations

Microbiology and histopathology examinations were immediately performed on the partial implants and surrounding tissue from the left breast (16 September 2011). No other specimens were sent for microbiological testing. On histopathology examination, breast implants were found to be surrounded by fibrous capsules and infiltrated with inflammation cells and phagocytosis by giant cells and capillary hypertrophy was also observed, which indicated foreign-body reaction. However, no solid evidence of bacterial or fungal infection was found.

In the meantime, bacterial culture was performed on the partial implants and tissue by inoculating them on Columbia agar supplemented with 5% sheep blood, China-blue lactose agar and chocolate agar. However, no fungal culture was performed initially as per surgeon's instructions. No bacteria were recovered. However, a notable amount (from the first to the second sector of the streaked plate) of yeast-like colonies were observed on Columbia blood agar on day 4 of incubation. Preliminary microscopic examination of the colonies showed yeast-like cells with a sympodial conidiogenesis. One pure colony of the isolate was then inoculated onto a chromogenic medium (CHROMagar *Candida*, CHROMagar Company, Paris, France) for identification, and was assigned as “*Candida tropicalis*” on day 8 based on the production of dark blue pigments. However, the patient had been discharged before the microbiology results were finalized.

### 4. Sequence-based identification

The above “*C. tropicalis*” strain was included in the CHIF-NET surveillance study (strain ID no. 11PU348). Genomic DNA was extracted by beating a fungal suspension with glass beads as described before [15]. Amplification of the fungal internal transcribed spacer (ITS) region and the D1/D2 domain of the 26S rRNA gene was performed as previously described with primer pairs ITS1/ITS4 and F63/R635, respectively [15]–[17]. The PCR products were sequenced in both directions using corresponding PCR amplification primer pairs at Ruibiotech Co. Ltd. (Beijing, China) using the DNA analyzer ABI 3730XL system (Applied Biosystems, Foster City, CA). Species identification was performed by comparing the obtained ITS and D1/D2 sequences against those in the Centraalbureau voor Schimmelcultures (CBS) Fungal Biodiversity Center database and GenBank using the BioloMICSNet and BLASTn software, respectively. A sequence similarity of 97% and 99% was applied as species identification ‘cut-off’ value for the ITS region and D1/D2 domain, respectively [18].

### 5. Phylogenetic analysis

All *Q. cyanescens* ITS and D1/D2 nucleotide sequences available in GenBank till 15 November 2013 (34 and 20 sequences for the ITS region and D1/D2 domain, respectively, Table 1) were compiled. Phylogenetic analysis was performed with software MEGA (Molecular Evolutionary Genetic Analysis software, version 6.0) using the Neighbor-Joining (NJ) method [19], [20], with all positions containing gaps and missing data eliminated from the data set. The significance of the cluster nodes was determined by bootstrapping with 1,000 randomizations. The evolutionary distances were computed using the Maximum Composite Likelihood method [21] and were in the units of the number of base substitutions per site. In addition, the ITS and D1/D2 sequences of *Q. coyrecup* WAC12947 (GenBank accession no. DQ823444.1 and DQ823431.1) [9], *Q. eucalypti* CMW1101 (DQ317625.1 and DQ317618.1) [8], *Q. pitereka* CMW6707 (DQ317627.1 and DQ317620.1) [8], *Q. simpsonii* CBS124772 (GQ303290.1 and GQ303321.1) [7] and *M. juglandis* KR0015442 (EU069498.1 and EU069497.1) [22] were downloaded for phylogenetic comparison (Table 1).

### 6. Antifungal susceptibility testing

Minimum inhibitory concentrations (MICs) of *Q. cyanescens* 11PU348 to fluconazole, voriconazole, itraconazole, posaconazole, caspofungin, micafungin, anidulafungin, 5-flucytosine and amphotericin B were determined *in vitro* by broth microdilution methods as per Clinical and Laboratory Standards Institute (CLSI) M38-A2 guidelines [23]. *Candida parapsilosis* ATCC 22019 and *Candida krusei* ATCC 6258 were used as the quality control strains for the test [23].

### 7. Nucleotide sequence accession numbers

The ITS region and D1/D2 domain sequences of strain 11PU348 were deposited in GenBank with accession numbers KF953496 and KF953497, respectively.

## Results

### 1. Sequence-based identification

By querying ITS region and D1/D2 domain sequences against those in the CBS database, the ITS region and D1/D2 domain sequences of *Q. cyanescens* 11PU348 showed 99.3% (576/580 bp) and 100% (600/600 bp) similarity to the ITS and D1/D2 sequences of *Q. cyanescens* type strain CBS 357.73 (GenBank accession number DQ119135.1 and DQ317615.1, respectively).

### 2. Phylogenetic analysis

**Figure 1:**
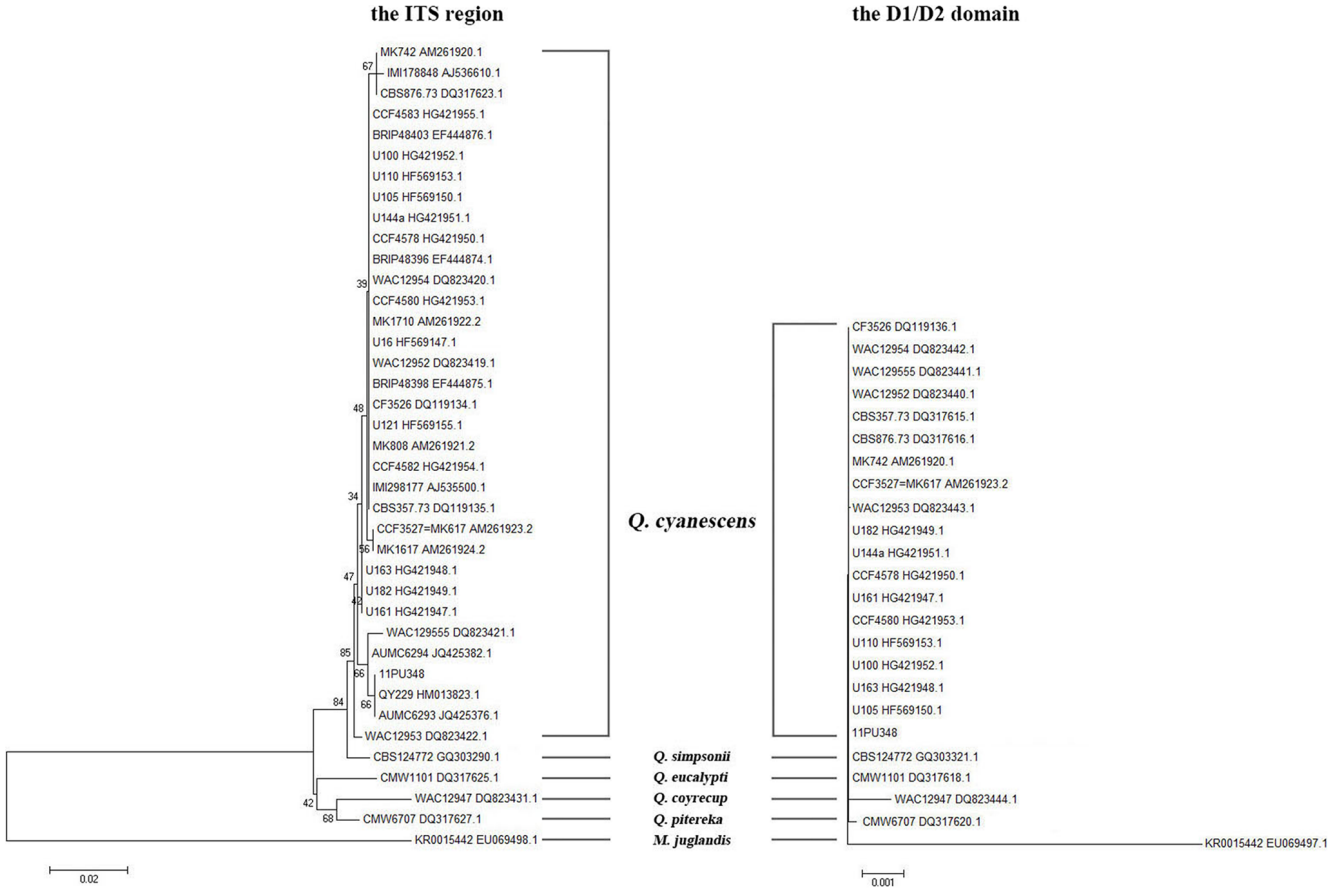
The Neighbor-Joining (NJ) tree of *Q. cyanescens* isolate 11PU348, all *Q. cyanescens* isolates with ITS and/or D1/D2 sequences available in GenBank, and selected isolates of other four *Quambalaria* species and *Microstroma juglandis*.

The nucleotide sequence alignments within *Q. cyanescens*, using sequences of *Q. cyanescens* type strain CBS 357.73 as references, showed this species with little inter-species variation within both the ITS region (99.0% to 100%) and D1/D2 domain (99.6% to 100%) (Table 1). Of note, the ITS region can clearly discriminate *Q. cyanescens* and other four *Quambalaria* species, with highest sequence similarity of less than 97.0%. However, the D1/D2 domain was not able to identify the five species within *Quambalaria* genus (sequence similarity >99.0%). The NJ analysis of the ITS region and D1/D2 domain yielded similar results (Figure 1).

### 3. Phenotypic characteristics on agar

**Figure 2:**
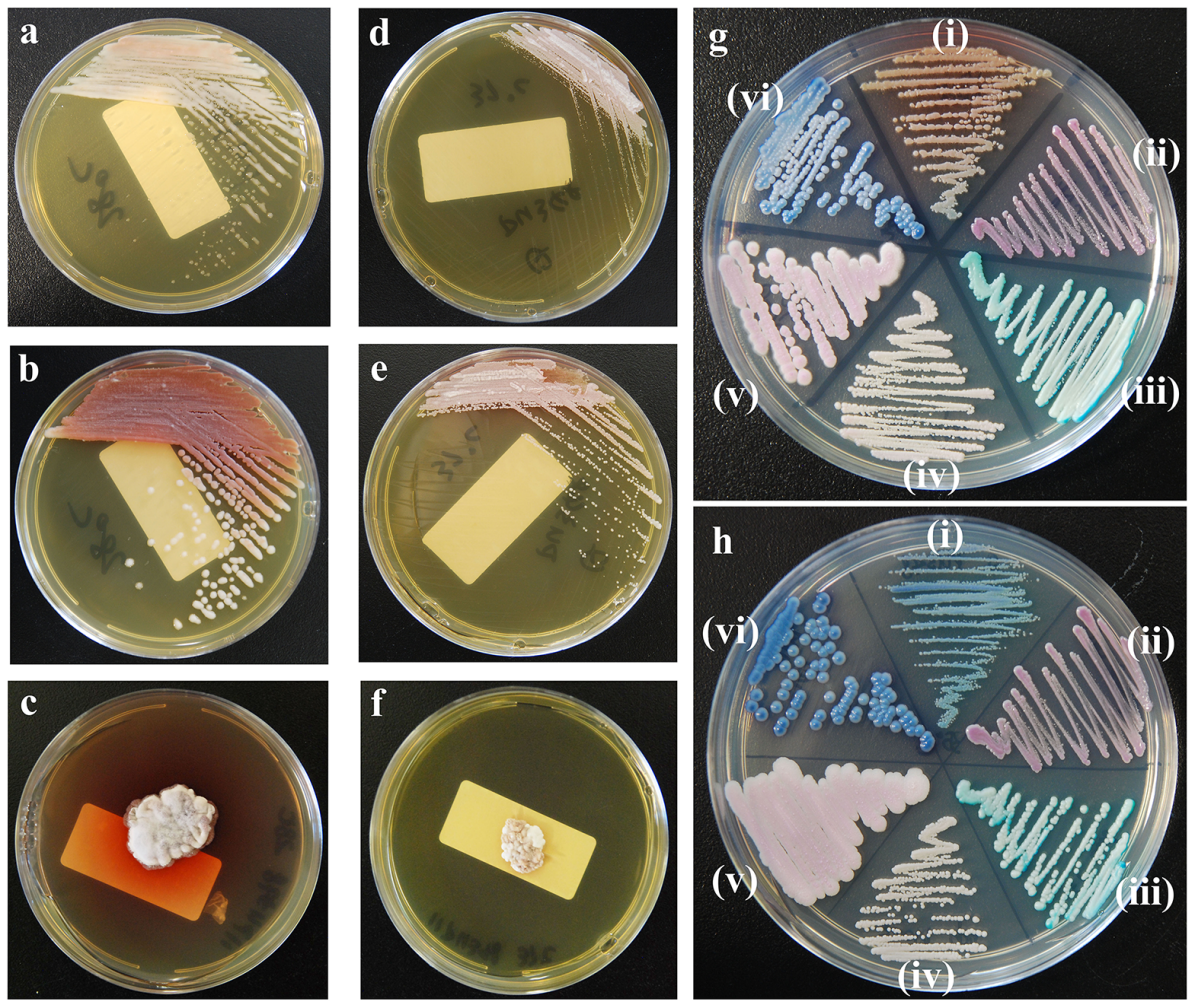
Phenotype of *Quambalaria cyanescens* 11PU348 on Sabouraud dextrose agar (**Figure 2a to 2f**) and CHROMagar *Candida* (**Figure 2g and 2h**). Incubation conditions: 2a and 2g, 28°C, 48 h; 2b, 28°C, 72 h; 2c, 28°C, 2 weeks; 2d and 2h, 37°C, 48 h; 2e, 37°C, 72 h; 2f, 37°C, 2 weeks. Strains used in Figure 2g and 2h: (i) *Q. cyanescens* 11PU348; (ii) *C. glabrata* sensu stricto 10H1043; (iii) *C. albicans* ATCC 90028; (iv) *C. parapsilosis* sensu stricto ATCC 22019; (v) *C. krusei* ATCC 6258; (iv) *C. tropicalis* 10H1048. *C. glabrata* sensu stricto 10H1043 and *C. tropicalis* 10H1048 were selected from CHIF-NET study [15].

*Q. cyanescens* isolate 11PU348 grew well at 28°C and 37°C, but failed to grow at 42°C on Sabouraud dextrose agar. By three-sector streaking on Sabouraud dextrose agar, the strain had yeast-like colonies which were initially moist, smooth, of various sizes and white colored within 48 h at 28°C (Figure 2a), and turned to be creamy, butyrous and exuding dark-orange pigment after 72 h incubation (Figure 2b). However, the strain grew slower when incubated at 37°C, and tended to be mold-like, especially in the first sector of the streaked plates (Figure 2d and 2e).

After more than 2 weeks' incubation at either 28°C or 37°C, a pure culture of the organism yielded a typical filamentous fungi phenotype that appeared to be restricted, velvety, furrowed, compact and cerebriform, accompanied by a red pigment and a burgundy reverse color. The production of pigments was more obvious at 28°C than at 37°C (Figure 2c and 2f).

On CHROMagar *Candida*, the colonies of *Q. cyanescens* 11PU348 were dark blue hybridizing with white, which was very similar to the phenotype of *C. tropicalis* when incubated at 37°C for 48 h (Figure 2h), but generating dark-orange pigment when incubated at 28°C (Figure 2g).

### 4. Microscopic morphology

Yeast-form of *Q. cyanescens* 11PU348 showed the typical sympodial conidiogenesis, and had smooth-walled, obovoidal, solitary or bearing secondary conidia. The filamentous form of the strain showed hyphae which were regular, hyaline, smooth-walled, branched and suberect. The conidia formed by sympodial growth of the conidiogenous cells (primary conidia) mostly give rise to several secondary conidia.

### 5. Antifungal susceptibilities

*Q. cyanescens* isolate 11PU348 exhibited high MICs to 5-flucytosine (MIC >64 µg/ml) and all echinocandins tested, including anidulafungin (MIC >8 µg/ml), micafungin (MIC >8 µg/ml) and caspofungin (MIC = 8 µg/ml). However, the isolate appeared more susceptible to amphotericin B (MIC = 0.5 µg/ml) and azoles (MICs of fluconazole, voriconazole, itraconazole and posaconazole were 0.5 µg/ml, <0.008 µg/ml, <0.015 µg/ml and 0.015 µg/ml, respectively).

## Discussion

*Q. cyanescens* is rarely identified in the clinical microbiology laboratory, and its pathogenic role is still uncertain. A review of literature shows that this fungus was recovered primarily from individuals who were immunocompromised or debilitated [11], [14], including a possible pulmonary case in a heart transplant patient [14], fungemia in lymphoma patients [11]. However, none of the above studies provided unequivocal clinical evidence of infection. In addition, Jackson *et al.* reported a pseudo-epidemic of *Q. cyanescens* pneumonia in a US hospital introduced by contamination of bronchoscopy suites [13], which suggests that the species may be an environmental contaminant in human patients.

Furthermore, fungal infections due to augmentation mammoplasties are rare. To date, only 15 out of 21 cases of breast implant fungal infections have been reported [24]–[37] (Table 2). *Aspergillus*, *Candida*, *Curvularia*, *Paecilomyces*, *Penicillium*, and *Trichosporon* spp. were potential causative agents. Most of the cases were efficaciously managed with implant removal, but some patients recovered after intravenous antifungal therapies (Table 2).

**Table 2:** Fungal infections in patients after augmentation mammoplasty previously reported.

Species	No. of cases	Country reported	Duration (mammoplasty to infection)	Implant removal	Antifungal therapy	Reference
*Candida albicans*	1	Italy	3 years	No	Caspofungin	[25]
*Candida albicans*	1	Turkey	5 years	Yes	Not specified	[32]
*Candida albicans*	1	US	4 years	Yes	Fluconazole	[29]
*Candida albicans*	1	US	10 months	Yes	Not specified	[34]
*Candida parapsilosis*	1	US	16 days	Yes	Fluconazole	[26]
*Trichosporon beigelii*	1	US	16 months	No	Fluconazole	[30]
*Trichosporon spp.*	1	Singapore	17 months	No	Fluconazole	[33]
*Aspergillus flavus*	1	UK	18 months	Yes	Not specified	[36]
*Aspergillus flavus*	1	US	4 years	Yes	Not specified	[31]
*Aspergillus niger*	1	UK	5 years	Yes	Not specified	[24]
*Aspergillus niger*	1	US	Several months	Yes	Not specified	[35]
*Curvularia spp.*	5	US	4–12 months	Not specified	Not specified	[27]
*Curvularia* spp.	1	US	6 months	Yes	Not specified	[34]
*Paecilomyces variotii*	1	US	14 months	Yes	Not specified	[37]
*Penicillium*	3	US	Not stated	Not specified	Not specified	[28]

We note that the pathogenic role of *Q. cyanescens* in this clinical case is questionable. There was no corroborating direct microscopic, histopathologic or serological evidence of fungal infection. Although the isolation was obtained from a specimen which showed histological signs of acute inflammation, this could be due to either real infection or foreign-body reaction. In addition, no samples (except routine bloods) other than the implant and the surrounding tissue removed during surgery were sent for laboratory examination, nor was repeat isolation attempted, as the present study was done retrospectively. The patient fully recovered after removal of implants, without any antifungal therapy administered. Although no other micro-organisms were isolated from this patient, and no fungal organisms were isolated from other patients who underwent plastic surgery during the same time-period, the possibility of environmental contamination cannot be excluded.

If this described case was due to a real infection, the slow progression of the inflammation, and the fact that the patient was both afebrile and asymptomatic with all blood test results within normal values, is consistent with an infection caused by a low virulence micro-organism. A previous experimental study in a murine model demonstrated that *Q. cyanescens* does have a low virulence potential [11].

Misidentification of *Q. cyanescens* 11PU348 was noted during confirmative identification process in CHIF-NET study [17]. Initially, two matrix-assisted laser desorption ionization–time of flight mass spectrometry systems (Vitek MS, bioMérieux, Marcy l'Etoile, France; Bruker Biotyper, Bruker Daltonics, Bremen, Germany) failed to identify strain 11PU348. Subsequent ITS sequencing identified the strain as *Q. cyanescens*. The main reason for the misidentification in the initial identification (that reported to clinic) was the yeast-like colonies with dark blue appearance at 48 h on CHROMagar *Candida* at 37°C, which was very similar to the appearance of *C. tropicalis* (Figure 2h). Although the patient in this case was cured by removal of the breast implant, the high MICs to 5-flucytosine and all echinocandins of *Q. cyanescens* were notable. Therefore, accurate identification of *Q. cyanescens* is important to avoid ineffective antifungal treatment. Mass spectra data of *Q. cyanescens* were neither represented in Vitek MS nor in Bruker Biotyper identification databases. Hence both MALDI-TOF MS systems assigned “no identification” to this isolate and importantly, did not misidentify the strain to another species. Although we were not able to identify *Q. cyanescens* against the current commercially available library of spectra, our result will nevertheless contribute to the existing spectral building.

In the most recent study, the ITS and D1/D2 sequences were used to cluster the *Quambalaria* genus and replaced *Q. cyanescens* species from another genus [8]. But in the present study, we found that the D1/D2 domain was not able to distinguish the different species within *Quambalaria* genus (Figure 1; Table 1). Compared with the D1/D2 domain, the ITS region was accurate in the identification of *Q. cyanescens* and other species within this genus (Figure 1; Table 1).

## Conclusions

In conclusion, *Q. cyanescens* is a rare clinical basidiomycetous pathogen. Here we report a *Q. cyanescens* strain isolated from a patient after augmentation mammoplasty in China. The possibility of its real pathogenic role was discussed. The high MICs to 5-flucytosine and all echinocandins highlight the importance of accurate identification so that appropriate therapy can be prescribed. To date, ITS sequencing remains the only available method to obtain an accurate identification result on this organism, while the pathogen is potentially misidentified as *C. tropicalis* by CHROMagar *Candida*.
